# Human attachment site preferences of ticks parasitizing in New York

**DOI:** 10.1038/s41598-022-25486-7

**Published:** 2022-12-03

**Authors:** Charles Hart, Laura A. Schad, Jahnavi Reddy Bhaskar, Erin S. Reynolds, Christopher P. Morley, Saravanan Thangamani

**Affiliations:** 1 Department of Microbiology and Immunology, Upstate Medical University, Syracuse, NY 13210 USA; 2 SUNY Center for Vector-Borne Diseases, Upstate Medical University, Syracuse, NY 13210 USA; 3 Institute for Global Health and Translational Sciences, Upstate Medical University, Syracuse, NY 13210 USA; 4 Department of Public Health and Preventive Medicine, Upstate Medical University, Syracuse, NY 13210 USA; 5 Department of Family Medicine, Upstate Medical University, Syracuse, NY 13210 USA; 6 Department of Psychiatry and Behavioral Sciences, Upstate Medical University, Syracuse, NY 13210 USA

**Keywords:** Ecology, Microbiology, Medical research

## Abstract

Ticks transmit several arthropod-borne pathogens in New York State. The primary human-biting ticks in this region are *Ixodes scapularis*, *Amblyomma americanum*, and *Dermacentor variabilis*. Body regions where tick bites human vary depending on the tick species and life stage, and clothing worn by the host. A community tick submission system was used to acquire information about bite-site location prior to pathogen testing to understand species and life stage-specific body-segment preferences. These data resulted in the identification of species-specific preferences for location, with *D. variabilis* preferentially biting the head and neck and *A. americanum* preferring the thighs, groin, and abdomen. *Ixodes scapularis* was found across the body, although it showed a significant life stage difference with adults preferring the head, midsection, and groin, while nymphs/larvae preferred the extremities. Infection with *Borrelia burgdorferi* resulted in a significant change in attachment site. This provides an assessment of which body region ticks of the most common species in New York are likely to be found.

## Introduction

Ticks are the primary vector of arthropod-borne pathogens in temperate environments. In New York state, ticks are primarily active between March and November, although cold-tolerant species are active in winter during intermittent periods of above-freezing temperatures^1,2^. The vast majority of ticks found feeding upon humans in New York State are *Ixodes scapularis*, *Dermacentor variabilis*, and *Amblyomma americanum*^1^.

*Ixodes scapularis* is the most common tick species encountered by humans in New York State^1^ and throughout New England, the Upper Midwest, and much of the northeastern United States. This tick species is an established vector of numerous pathogens, including *Borrelia burgdorferi*, *Anaplasma phagocytophilum*, *Babesia microti*, *Borrelia miyamotoi*, Deer Tick virus (Powassan virus Lineage 2), *Ehrlichia muris eauclairensis *^3–8^, and potentially undiscovered pathogens.

*Amblyomma americanum* is associated with a more southern range of the United States, but has been recently expanding into the Northeast, including New York^2^. They may bite various vertebrates at any life stage, with humans being a potential target for all three life stages. This tick transmits *Ehrlichia chaffeensis,* unknown *Borrelia* species that is associated with Southern Tick-Associated Rash Illness (STARI), *Francisella tularensis,* Heartland virus and Bourbon virus^9–13^. The saliva of these ticks, even when uninfected, can also cause an allergy to galactose-alpha-1,3-galactose (alpha-gal)^14^, resulting in a potentially severe acquired food allergy to red meat.

*Dermacentor variabilis* is common throughout the United States^2^. The ticks are commonly associated with dogs and domestic animals. This tick species is an established vector of *Francisella tularensis*^15^ and *Rickettsia rickettsii*^16^. The saliva of uninfected *D. variabilis*, however, may cause flaccid paralysis in children due to a neurotoxic salivary protein^17^.

The location of tick attachment is of clinical importance because it can allow for ticks to be rapidly discovered and removed, curtailing their ability to transmit pathogens. Here, we describe analysis of our survey data gained from a community-engaged passive surveillance program^1^ to identify the regions of the body where ticks are most likely to be found in relation to tick species and whether or not the presence of tick-borne pathogens impact attachment region selection. We provide evidence that infection of *I. scapularis* with *Borrelia burgdorferi* resulted in a significant change in tick attachment site. This can be applied to tick protection or clinically to inform the search for biting ticks or cutaneous signs of tick-borne illness such as erythema migrans or eschar.

## Results

### Ticks submitted from humans were from three primary species

Ticks were submitted by the public between April and December 2020 with an associated questionnaire indicating whether the ticks were collected from humans and from which body part they were found. In total, 1743 ticks (1266 adult, 477 nymphs/larvae) were submitted from humans (Table 2). These included 1408 *I. scapularis* (1066 adult, 342 nymphs/larvae), 190 *A. americanum* (56 adult, 134 nymphs/larvae) and 145 *D. variabilis* (144 adults and one nymph). The tick most commonly encountered biting humans, therefore, was *I. scapularis*, followed by *A. americanum* and *D. variabilis* to a lesser but still noticeable extent.

### Human location preference is species-specific

**Figure 1:**
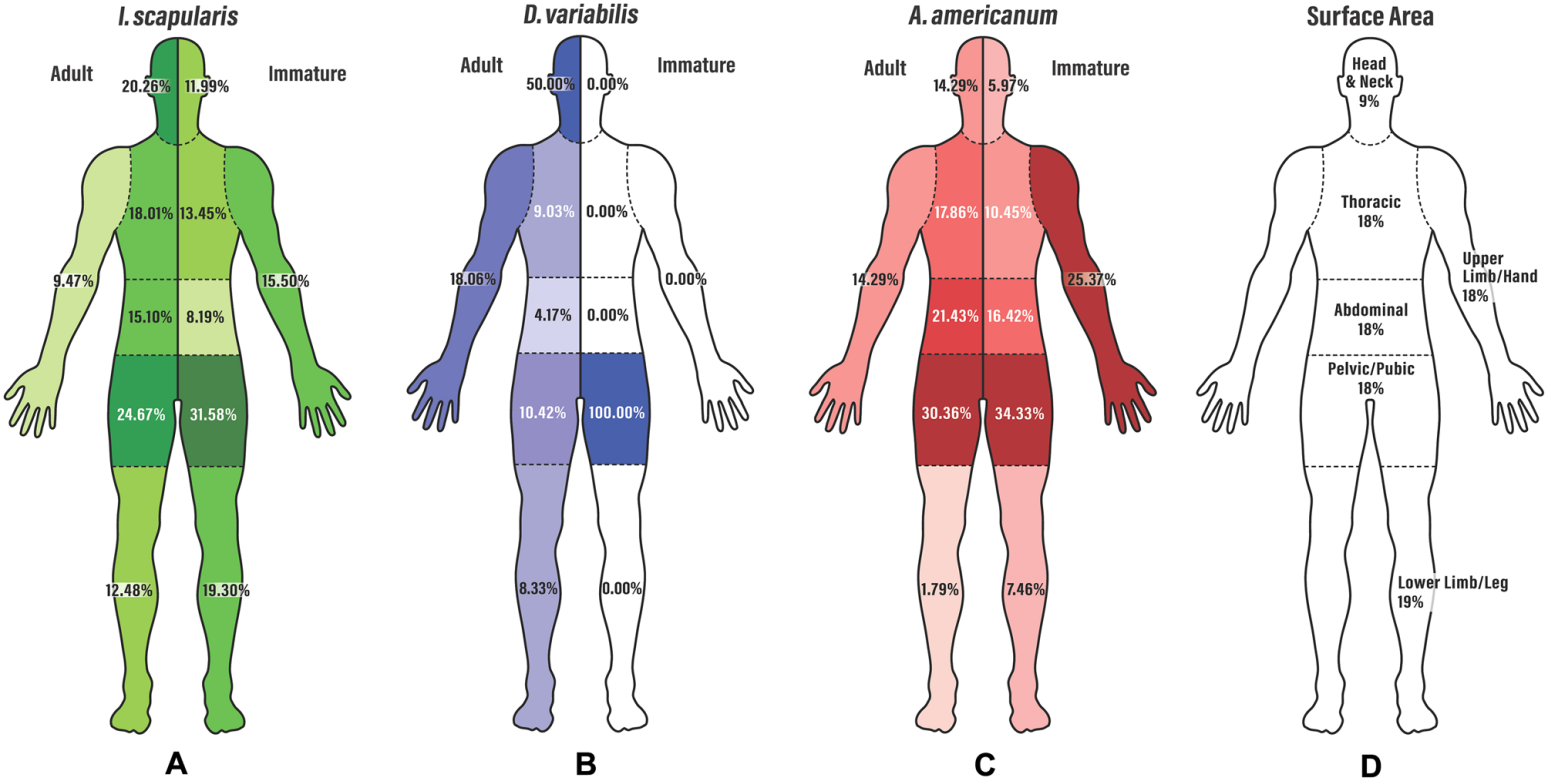
A representation of the body segment distribution of (**A**) *I. scapularis*, (**B**) *D. variabilis*, and (**C**) *A. americanum*. These are divided into adult and nymph/larval ticks for each species. These are compared to the corresponding average predicted surface area of each bodily segment (**D**). Statistically significant changes in distribution were observed compared to the assumption of equal distribution over the body, with *D. variabilis* strongly preferring the head and neck and *A. americanum* preferring the groin/pelvic/thigh area, with *I. scapularis* showing a more somewhat even distribution. Figures were generated with assistance from Sabra Snyder.

For ticks found on humans, the body locations listed in the questionnaire data were aggregated into six categories: the head/neck, thoracic region, abdominal region, pelvic/pubic region, upper limbs, and lower limbs. This condensation is depicted in Table 1. In addition, the location preferences of three species of any life stage (*I. scapularis*, *D. variabilis*, and *A. americanum*) were compared, separating adult ticks from immature life stages consisting of the combined number of nymphs and larvae. The results are displayed in Fig. 1A–C and further described in Table 2.

**Table 1:** Assignment of body part (n = 21) to condensed categories of body segment (n = 6).

Body part	Body segment
Scalp	Head and neck
Face	
In/around ears	
Neck	
Chest	Thoracic
Armpit	
Upper back	
Stomach	Abdominal
Inside belly button	
Lower back	
Groin	Pelvic/pubic
Thigh	
Waist/hip	
Hand	Upper limb (hand)
Lower arm/wrist	
Upper arm	
Shoulder	
Behind knee	Lower limb (leg)
Calf/shin	
Ankle	
Foot

**Table 2:** Body segment where ticks were found, by life stage, stratified by tick species. Statistical comparisons were conducted to compare lifestage attachment, calculated for each body segment.

	Adult N (%)	Nymph/larvae N (%)	χ^2^	*p*
***I. scapularis***				
Head and neck	216 (20.26)	41 (11.99)	11.881	.001*
Thoracic	192 (18.01)	46 (13.45)	3.835	.050*
Abdominal	161 (15.10)	28 (8.19)	10.657	.001*
Pelvic/pubic	263 (24.67)	108 (31.58)	6.366	.012*
Upper limb/hand	101 (9.47)	53 (15.50)	9.641	.002*
Lower limb/leg	133 (12.48)	66 (19.30)	9.929	.002*
Total	1066	342		
***A. americanum***				
Head and neck	8 (14.29)	8 (5.97)	3.541	.060
Thoracic	10 (17.86)	14 (10.45)	1.965	.161
Abdominal	12 (21.43)	22 (16.42)	0.675	.441
Pelvic/pubic	17 (30.36)	46 (34.33)	0.281	.596
Upper limb/hand	8 (14.29)	34 (25.37)	2.820	.093
Lower limb/leg	1 (1.79)	10 (7.46)	2.334	.179^
Total	56	134		
***D. variabilis***				
Head and neck	72 (50.00)	0 (0.00)	N/A	N/A
Thoracic	13 (9.03)	0 (0.00)	N/A	N/A
Abdominal	6 (4.17)	0 (0.00)	N/A	N/A
Pelvic/pubic	15 (10.42)	1 (100.00)	8.118	.110^
Upper limb/hand	26 (18.06)	0 (0.00)	N/A	N/A
Lower limb/leg	12 (8.33)	0 (0.00)	N/A	N/A
Total	144	1		

*Denotes significance at *p* = 0.05 level.

^Fisher’s Exact.

These values were compared against the number of ticks present on each area of the body, assuming an equal distribution based on the surface area of the human body (Table 2). The surface area of the body was estimated based on burn assessment charts, modified to provide a position for the groin/pelvic region consisting of 10% (Fig. 1D). A statistical difference versus even distribution was identified.

For *I. scapularis*, the bodily distribution of ticks was relatively even, with a distribution with more ticks found in the groin/pelvic region (20.26% adult, 11.99% nymphs/larvae), the abdominal section (15.10% adult, 8.19% nymphs/larvae), the thoracic region (18.01% adult, 13.45% nymphs/larvae) and the head (20.26% adult, 11.99% nymphs/larvae). Despite the large surface area of the legs, comparatively few ticks were found there, at 12.48% adult and 19.30% nymphs/larvae versus a surface area of 19%. This is also true for the arms, with 9.47% of adult ticks and 15.50% of nymphs/larvae ticks distributed over an expected 18% surface area. The biting preference of adult and immature ticks (larvae and nymphs) were compared for each species using Chi-squared tests and is displayed in Fig. 1A.

A more extreme version of this tendency is observed with *A. americanum*, which strongly prefers the midsection, especially the groin/pelvic region and abdominal area. For these ticks, 30.36% of adult and 34.33% of nymphs/larvae ticks were found attached to the groin/pelvic region, including the thighs. Comparatively few were found on the lower legs (1.79% adult, 7.46% nymphs/larvae) and head (14.29% adult, 5.97% nymphs/larvae), demonstrating that these ticks have a strong tendency to bite quickly and in areas covered with clothing.

In contrast to *I. scapularis* and *A. americanum*, *D. variabilis* was found to have a strong tendency to attach to the head and neck region. Despite containing 9% of the surface area of the human body, the head was identified as the attachment point of 50% of all *D. variabilis* found on humans. In contrast to the other two species, these ticks prefer to climb and are not encouraged to feed by the presence of clothing. Rather, they show a strong preference for the scalp. The one submitted nymph was from the groin/thigh area, although this sample size is too small to relate to the overall behavior of immature *D. variabilis*.

### Life stage influences tick attachment location

The biting preference of adult and immature ticks (larvae and nymphs) were compared for each species using Chi-squared tests and displayed in Fig. 1A.

For *I. scapularis*, a statistically significant difference in attachment location was observed between adult and nymph/larval ticks for all body segments (Table 2). As shown in Fig. 1A, and Table 2, adults showed a stronger preference for the head and neck (20.26%) versus nymphs and larvae (11.99%). This pattern additionally holds true for the thoracic region, which was more often bitten by adults (18.01%) than immature life stages (13.45%). In contrast, however, nymphs and larvae were more likely to be found on the upper limbs (15.50% versus 9.47% of adults) and the lower extremities (19.30% versus 12.48% for adults). Additionally, they were found less commonly in the abdominal region (8.19% versus 15.10% for adults) and more often in the pelvic/pubic region (31.58% versus 24.67% for adults). This suggests that nymph and larval *I. scapularis* are less restricted to the central portion of the body, are more identified on the limbs, and are more likely to bite more rapidly after host contact (targeting the legs and groin more often).

For *A. americanum*, no statistically significant difference in attachment point was observed between adult and nymph/larval tick populations on any body region. Most *A. americanum* found on the upper limbs consist of nymphs/larvae (25.37%) versus adults (14.29%), likely due to the high visibility of adult *A. americanum*. However, the overall behavior in all life stages is similar, with a preference for the pelvic/pubic region (30.36% for adults, 34.33% for nymphs/larvae), indicative of a rapid biting response in areas of the body that are covered by clothing.

Statistical significance could not be determined for *D. variabilis*. This is due to the remarkably low rate of *D. variabilis* nymph/larvae submission, as noted previously^1^. In addition, *Dermacentor variabilis* is almost exclusively encountered by humans in its adult life stage, with nymphs and larvae are rarely observed on either humans or domestic animals.

### Pathogen infection of *I. scapularis* alters body segment preference

Of the three-primary species of tick submitted, only *I. scapularis* was determined to contain medically significant pathogens. However, of the ticks found on humans, several pathogens were noted, with the principal members being *Borrelia burgdorferi*, *Anaplasma phagocytophilum*, and *Babesia microti*, as well as a group for other pathogens including *B. miyamotoi* and Deer Tick virus, which were only observed in a small percentage of the total *I. scapularis*. The numbers of ticks containing these pathogens associated with each body region are listed in Table 3.

**Table 3:** *Ixodes scapularis* (deer tick) pathogen infection rate organized by body segment attachment (n = 1408).

	*Borrelia burgdoferi*	*Anaplasma phagocytophilum*	*Babesia microti*	Other	None
	N (%)	N (%)	N (%)	N (%)	N (%)
Head and neck	58 (15.4)	21 (17.8)	34 (21.9)	5 (27.8)	156 (17.9)
Thoracic	75 (19.9)	26 (22.0)	29 (18.7)	7 (38.9)	129 (14.8)
Abdominal	56 (14.9)	15 (12.7)	18 (11.6)	2 (11.1)	117 (13.4)
Pelvic/Pubic	109 (28.9)	35 (29.7)	32 (20.6)	2 (11.1)	228 (26.2)
Upper Limb/Hand	29 (7.7)	11 (9.3)	15 (9.7)	0 (0.0)	110 (12.6)
Lower Limb/Leg	50 (13.3)	10 (8.5)	27 (17.4)	2 (11.1)	131 (15.0)
TOTAL	377	118	155	18	871
χ^2^ (*p* value*)*	12.347 (0.030)	5.830 (0.323)	5.790 (0.327)	8.560 ^#^(0.087)	13.394 (0.020)

The “other” category consists of the combined numbers of low-rate pathogens including *B. miyamotoi* and Deer Tick virus.

^#^Fisher’s Exact.

**Figure 2:**
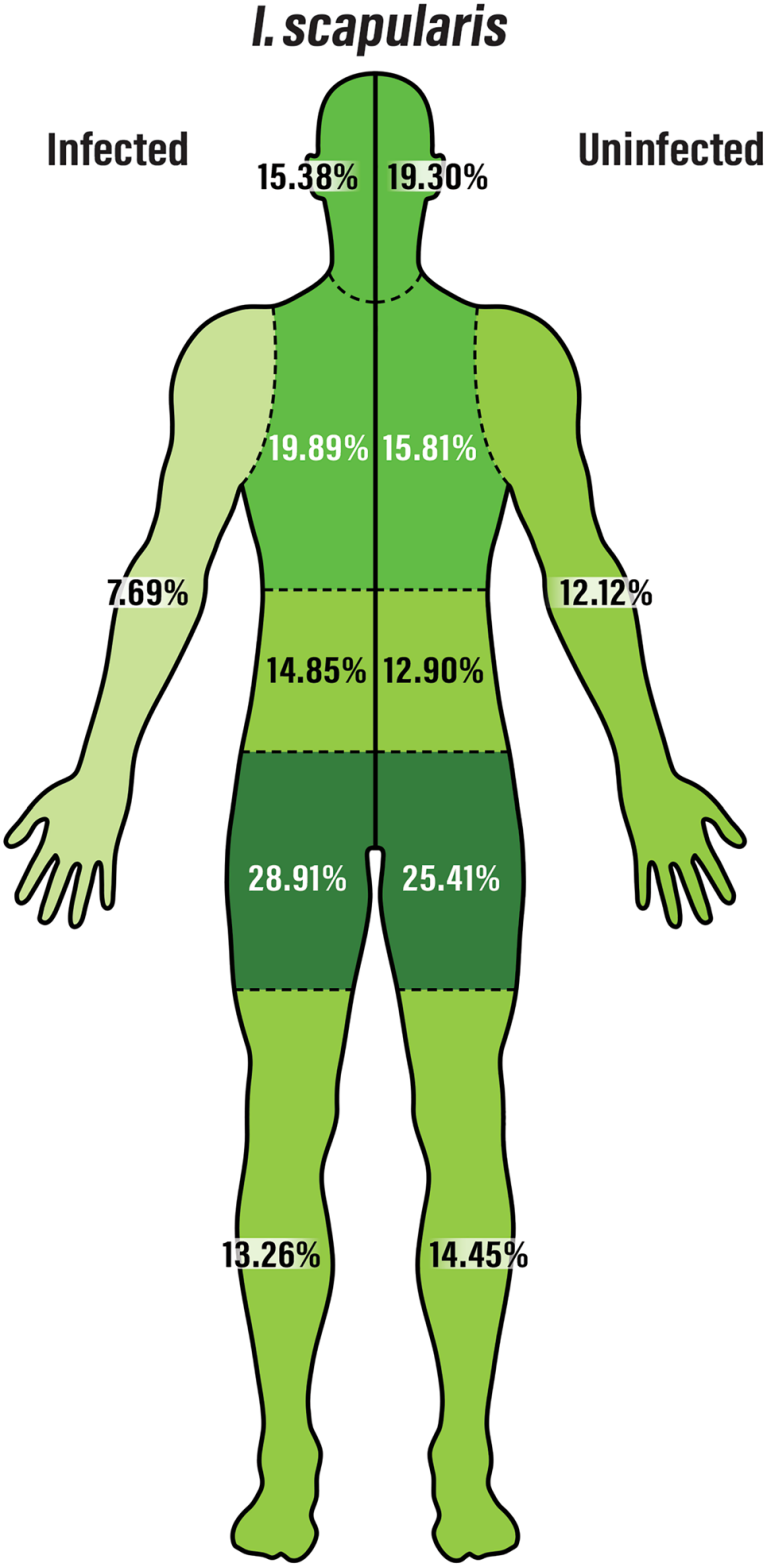
A representation of the body segment distribution of pathogen-infected *I. scapularis* versus uninfected *I. scapularis*, indicating an increased preference toward the limbs and head. This effect is related most strongly to infection with *B. burgdorferi*. The figure was generated with assistance from Sabra Snyder.

The number of ticks on body regions with and without specific pathogens was compared using Chi-squared tests. No statistically significant change in bodily distribution was observed between uninfected ticks and ticks infected with *A. phagocytophilum* (p = 0.323), *B. microti* (p = 0.327), or other pathogens (p = 0.087). However, statistical differences were observed for ticks infected with *B. burgdorferi* (p = 0.030) and ticks with no detected pathogens (p = 0.020). The biting preference of pathogen-infected *I. scapularis* is displayed in Fig. 2.

For ticks infected with *B. burgdorferi*, this alteration in biting behavior resulted in a decreased tendency to bite the head (15.38% for *B. burgdorferi*-infected ticks versus 19.30% for non-*B. burgdorferi*-infected ticks) and the upper limbs (7.69% versus 12.12%), with increased biting of the thoracic, abdominal, and pelvic/pubic areas. For non-*B. burgdorferi* ticks, there was an increase in biting of the upper limbs (12.12% versus 7.69%) and lower limbs (14.45% versus 13.26%) and a decrease in biting of the thoracic area (15.81% versus 19.89%).

## Discussion

The attachment site of ticks has been studied in the context of both animal and human tick preference. In Oklahoma, a study of horses indicated that *A. americanum* preferentially bites the inguinal area, while *I. scapularis* and *D. albipictus*, the moose-tick, primarily bite the chest and axillary region, with *D. albipictus* often being found on the back^18^. A survey of dogs and cats across the US identified a similar distribution of ticks on dogs, with the attachment being most common on the abdomen, axillary and inguinal regions. However, this was species-specific with *D. variabilis* preferring the head and neck specifically^19^. Cats were more successfully parasitized by *I. scapularis* which preferred the head and *A. americanum,* which preferred the tail and perianal region^19^. This is similar to a study of tick distribution on wild black bears (*Ursus americanus*) in Pennsylvania, indicating that the primary tick present was *I. scapularis* and that the greatest numbers were found in association with the ears and muzzle^20^. In these cases, the ability for ticks to attach to specific areas is most likely a result of the grooming habits and abilities of the animals in question.

Studies of anatomical region preference in humans also reported tick bite-site specificity associated with particular tick species. For example, in Korea, *H. longicornis* was determined to prefer abdomen and lower extremities (33%) and the abdomen/inguinal area (26.4%)^21^, which is a behavior similar to that of *A. americanum* observed here. Although *H. longicornis* is present in New York^1^, insufficient numbers were detected to draw definitive conclusions about its biting preference here. Additionally, a study in England (*I. ricinus*) reported that tick bites were most common in the legs (50%) of adult humans, but in the head and necks of children (43%)^22^, a differentiation that our survey does not at this time include. A similar phenomenon was observed in Russia, where tick bites were most common on the head and neck of all individuals (39.2%), but were much more common in children (84.9%)^23^. This study determined that the bite-site of single tick bites that resulted in infection with the Tick-Borne Encephalitis virus (TBEV) were associated with lethal outcomes if the bites were located on the head, neck, arms or axilla, while less lethality was associated with bites to the lower limbs and groin. This is most directly analogous to the transmission of DTV by *I. scapularis*, suggesting that bite site may have a similar relationship to disease outcomes in the related North American pathogen/vector pair.

Under normal circumstances, ticks exist in sylvatic cycles with specific host preferences based on the tick species and life stage, with spillover to humans occasionally occurring for species with generalist feeding habits. Therefore, the feeding behaviors of ticks are variable, and this influences the ways that the ticks interact with humans.

### Ixodes scapularis is less specific in host-site preference

The primary life stages of *I. scapularis* that bite humans include nymphs and adult females, although males may also be found on humans. The body segment preference of *I. scapularis* is less specific than for *D. variabilis*, which prefers the head, and *A. americanum*, which prefers the thighs and pelvic region. *Ixodes scapularis* is primarily found on the central trunk, including the groin/pelvic region, the abdomen, the thoracic region, and the head/neck. This varies between the life stages, with more adults found in the thoracic/abdominal region of the body and nymphs being more commonly found on the arms and legs. This is partly due to the substantial size difference between adult and nymph/larval *I. scapularis*, with larvae being almost imperceptible and nymphs having a total body length of two to four millimeters. This results in nymphs/larvae being much more difficult to see, allowing them to more readily attach to the most visible portions of the human body while adults are restricted mostly to areas covered by clothing and hair.

The presence of ticks on the head and neck indicates that *I. scapularis* tends to climb, although not with the preference for hair observed with *D. variabilis*. They appear to spend substantial time moving on the host, a period where they can be removed easily without having had a chance to potentially transmit pathogens by biting. On deer, this corresponds to a preference to move toward the neck and ears where the ticks are more difficult to dislodge^24,25^. On humans, it results in wide distribution across the whole body with less location specificity than other ticks.

In addition to body region and life stage identification, *I. scapularis* ticks were also screened for several pathogens to determine if infection status influences host site preference. *Anaplasma phagocytophilum*, *B. microti*, and other pathogens (DTV and *B. miyamotoi*) did not influence the body segment the ticks chose to feed. However, in ticks infected with *B. burgdorferi*, a statistically significant change in the distribution of tick bites marked by an increased report of tick bites in the midsection and a decreased tick bites in the arms, legs, and head. While this may suggest a change in tick behavior/fitness in response to infection, it may also relate to the differences in infection rates of adult and nymph/larval ticks. Larvae, having never fed, are not infected with *B. burgdorferi*, and the rate of infection in nymphs is lower than that of adults^1^. Nymphs are less likely to be infected and are more likely to attach to the arms and legs, which is a potential source of the observed difference in infection rates. However, it remains unclear why this is not observed for the other pathogens that follow the same trend of increased infection rate in adult versus nymph/larval ticks.

Bacterial and protozoal agents transmitted by *I. scapularis* take several hours for an infectious dose to be transmitted^26–28^. Therefore, prompt detection and removal of ticks is important for preventing tick-borne disease. Furthermore, understanding where the ticks attach allows them to be more easily detected, and also assists in preparing protective clothing for individuals entering tick-endemic areas. Additionally, knowing the biting location of *I. scapularis* could aid in detecting potential erythema migrans, a skin condition that occurs at the point of *B. burgdorferi* infected tick exposure in about 80% of cases^29^, which is highly diagnostic for both Lyme disease and STARI, which is transmitted by *A. americanum*.

### *Amblyomma americanum* prefers the thighs and groin of subjects

*Amblyomma americanum*, the lone star tick, is present throughout the southern portion of New York and is particularly dominant on Long Island^1^. This species is relatively large, fast, and aggressive, feeding on various animals, including deer, medium-sized animals, and birds^30^. As a generalist feeder, both adult and nymph/larval *A. americanum* often bite humans in endemic areas. This experiment identified six larvae, 107 nymphs, and 48 adult *A. americanum* from human sources. The dominance of nymph submissions is likely due to the large size of the tick, making nymphs and adults easier to spot in more visible areas.

In terms of body segment location, all life stages of *A. americanum* were most often found in the thigh/groin/pelvic region. Considering that most humans encounter ticks while walking through vegetation, the ticks most likely first adhere to the legs and move upward before biting. In this case, the ticks bite rapidly instead of ascending in large numbers to the torso or head. This area is also almost invariably covered in relatively tight-fitting clothing. The closeness of the fabric may also assist in inducing the ticks to feed by slowing their ascent and creating contact to induce biting.

While it does not transmit the same range of pathogens as *I. scapularis*, *A. americanum* is still a medically significant species. This species can transmit *Ehrlichia chaffeensis* and *E. ewingii*^31,32^, which are at present rare in New York, but are likely to increase as more *A. americanum* becomes established. *Amblyomma americanum* is also associated with Southern Tick-Borne Rash Associated Illness (STARI)^11^, a disease of unknown etiology that has previously been observed in New York^33^ and with galactose-alpha-1,3-galactose (alpha-gal) allergy, a reaction to the tick’s saliva that can result in a long term, potentially serious allergic sensitivity to the consumption of red meat. While the attachment time required to transmit or induce these pathogens is still unclear, prompt detection and removal of the tick is still recommended. Knowing the approach of the tick and where it is likely to be found improves this process.

Additionally, it is unclear if the results observed for *A. americanum* also apply to the related *A. maculatum*, the vector of *Rickettsia parkeri*, a cause of spotted fever. These ticks have been observed in the southernmost portions of New York with a high infection rate with *R. parkeri*^34^. Since early *R. parkeri* infection may result in a visible eschar, understanding where the eschar is most likely located can be critical for rapid diagnosis before the onset of severe disease symptoms. Considering the similarities in behavior between the two *Amblyomma* species, it may have similar preferences to *A. americanum*. Other escharotic diseases, such as *F. tularensis*, may also be present and linked to a tick with a highly dissimilar segment preference. The location of the escar itself, therefore, may be at least partially diagnostic for specific pathogens. However, at present, the sample size within this community engaged passive surveillance program is too small to assess its biting behavior in detail.

### *Dermacentor variabilis* exhibits preference for the human head

In this study, *D. variabilis* was almost exclusively encountered in its adult life stage. This indicates that while the adult ticks are generalist feeders that may bite humans, the nymph and larval stages are not and have much greater host specificity, either feeding exclusively on a specific type of animal or being restricted to the vicinity of animal burrows. The exact identity of the preferred larval and nymphal host of *D. variabilis* in New York could not be determined from these data, but is presumed to be one or several rodent species, lagomorph, or mesocarnivore with broad distribution across the eastern United States.

Additionally, *D. variabilis* was unique among the three species of ticks studied here. It had a strong bias toward the head and neck of human hosts, as opposed to a higher preference for the midsection and pelvis/groin with *I. scapularis* and especially *A. americanum*. This is clear evidence of climbing behavior, tending upward, but is also indicative of a strong preference for dense hair. In contrast to *I. scapularis* and *A. americanum*, *D. variabilis* in its adult stage is less likely to feed on deer^35,36^, with a preference for canids^36^, hence its colloquial name as the “American dog tick”. Hair provides the ticks with the same benefits as feeding on canids. It protects them from being immediately detected and removed, obscuring them until they can feed extensively. This can be of potential medical consequence in the case of tick paralysis, a condition of flaccid paralysis associated with the bite of *Dermacentor* spp. ticks^30^. In such cases, prompt removal of the tick is critical for treatment. Therefore, understanding its most likely location can be useful for removal of the tick before the onset of the condition, diagnostically to confirm the presence of the tick, or during treatment to ensure its removal. Considering that the tick will most likely be adult, it should be relatively obvious with careful observation.

### Limitations of this study

The data described in this manuscript derived from a set of ticks submitted by general public, with site location from a questionnaire completed upon tick submission. While speciation and pathogen testing were performed under laboratory conditions, the public completed the initial survey and is therefore subject to a level of inherent error and ambiguity. In the context of this study, this mainly concerns whether the body location submitted concerns an attachment or a tick that is still crawling over the potential host in preparation for biting. The term “attachment” may be colloquially interpreted as to contain both categories, or a person can potentially be mistaken about the state of the tick. While ticks filled with blood have fed, the situation is more indeterminate for short-duration attachments where the ticks have not yet begun to engorge. This may introduce some level of error from ticks found on a body segment that were not, at the time of collection, attached. However, the data are overall still useful for predicting the most likely location where ticks of specific species can be found on a person. Studies with test subjects and ticks under controlled conditions may assist in elucidating this matter further. Additionally, this data set was compiled without regard to gender and age group. This data was not collected with this version of the questionnaire; therefore, the tick attachment cannot be stratified by any demographic parameters of tick submitters.

## Conclusions

Three species of human-biting ticks (*Ixodes scapularis*, *Amblyomma americanum*, and *Dermacentor variabilis*) were submitted from a community-engaged tick submission system in New York State, indicating the bodily attachment location of the tick. From this, it was determined that *I. scapularis* is less specific in its preferential attachment sites. However, the life stage and infection status impact the tick's attachment site. The immature life stages prefer the limbs, while *B. burgdorferi* infected *I. scapularis* prefers to bite the central trunk versus the limbs. In contrast, *D. variabilis* prefers to attach to the head, and *A. americanum* prefers to attach to the lower midsection, upper legs, and groin.

This information is valuable for predicting the biting location of ticks dependent on species. It is also informative for the public to check for ticks at multiple anatomical regions, potentially reducing the transmission of tick-borne pathogens. This can then facilitate the quick removal of ticks to prevent possible pathogen introduction or pathogen testing of the tick for diagnostic considerations.

## Methods

### Tick collection and testing

As published previously, ticks were submitted from throughout New York State by the public as part of a Citizen-Science pathogen-screening effort^1^. Briefly, individuals from New York submitted ticks by mail in addition to an online questionnaire (Supplementary Fig. 1). Upon receipt, these ticks were identified morphologically to determine species and life stage and washed in 70% ethanol followed by deionized water. RNA extraction and pathogen detection were performed on these ticks as described^1^.

The tick submission survey information, information recorded on receipt, and pathogen status of each tick was stored in a Redcap^37,38^ database for storage and access of complete, filtered datasets. The tick database was restricted to ticks submitted between April and December 2020. The species on which the tick was found and, for ticks found on humans, the bodily locations of the ticks were collected from questionnaire data associated with each tick. The species and life stage of the ticks were also noted, selecting specifically the three most commonly observed tick species (*I. scapularis*, *D. variabilis*, and *A. americanum*). For I. scapularis, pathogen status was also considered, as no relevant pathogen rates were observed in *D. variabilis* and *A. americanum* that had been found on humans.

### Data preparation

Ticks were found on a reported 21 body parts. To simplify the analysis, body parts were condensed into six segments (Table 1). Three pathogens of interest were identified, and a binary Positive/Negative variable created for each. All remaining pathogens were grouped as “Other”, and no pathogen present was also created (both binary Positive/Negative). Three tick species were included in the dataset (*A. americanum, D. variabilis, I. scapularis*). Many tick samples were found to be carrying multiple pathogens (n = 119).

### Analysis

A crosstabulation of body segment by species and life stage was constructed to determine whether species and/or life stages appear to have body segment preferences. We calculated Pearson χ^2^ statistics (or Fisher’s Exact tests, where appropriate) to determine whether the observed distribution of tick life stages across body segments, by species, differed significantly from statistically expected proportional distributions, for *A. americanum* and *I. scapularis.* As only one *D. variabilis* nymph or larvae was included in the data set, statistical analysis of body part by life stage was not meaningful for this species. Additionally, A priori testing was conducted via crosstabulation of tick species against pathogen. *Ixodes scapularis* is the most significant vector of medically significant pathogens within New York State, and within this dataset, only *I. scapularis* was found to be infected with any pathogens (n = 1408) while all screened *A. americanum* and *D. variabilis* were negative for all tested pathogens. Therefore, Crosstabulation (with Pearson χ^2^ or Fisher’s Exact test) was employed to determine differences of body segment location by each pathogen status, specifically within *I. scapularis*. All analyses presented were conducted in SPSS v.28.

## Supplementary Information

Supplementary Information.

## Data Availability

All data generated or analysed during this study are included in this published article.

## References

[1] Hart CE, Bhaskar JR, Reynolds E, Hermance M, Earl M, Mahoney M, Martinez A, Petzlova I, Esterly AT, Thangamani S (2022). Citizen science tick surveillance as a public health tool to track the emergence of ticks and tick-borne diseases in New York. PLOS Glob. Public Health.

[2] CDC. *Regions where ticks live.* Centers for Disease Control and Prevention, viewed 30 November 2021. https://www.cdc.gov/ticks/geographic_distribution.html (2021).

[3] Hu LT (2006). Lyme disease. Ann. Intern. Med..

[4] Little EAH, Molaei G (2020). Passive tick surveillance: exploring spatiotemporal associations of *Borrelia burgdorferi* (Spirochaetales: Spirochaetaceae), *Babesia microti *(Piroplasmida: Babesiidae), and *Anaplasma phagocytophilum* (Rickettsiales: Anaplasmataceae) infection in *Ixodes scapularis* (Acari: Ixodidae). Vector Borne Zoonotic Dis..

[5] Johnson TL, Graham CB, Maes SE, Hojgaard A, Fleshman A, Boegler KA, Delory MJ, Slater KS, Karpathy SE, Bjork JK, Neitzel DF, Schiffman EK, Eisen RJ (2018). Prevalence and distribution of seven human pathogens in host-seeking *Ixodes scapularis* (Acari: Ixodidae) nymphs in Minnesota, USA. Ticks Tick Borne Dis..

[6] Pritt BS, Sloan LM, Johnson DKH, Munderloh UG, Paskewitz SM, McElroy KM, McFadden JD, Binnicker MJ, Neitzel DF, Liu G, Nicholson WL, Nelson CM, Franson JJ, Martin SA, Cunningham SA, Steward CR, Bogumill K, Bjorgaard ME, Davis JP, McQuiston JH, Warshaur DM, Wilhelm MP, Patel R, Trivedi VA, Eremeeva ME (2011). Emergence of a new pathogenic *Ehrlichia* species, Wisconsin and Minnesota, 2009. N Engl J Med..

[7] Telford SR, Armstrong PM, Katavolos P, Foppa I, Garcia AS, Wilson ML, Spielman A (1997). A new tick-borne encephalitis-like virus infecting New England deer ticks, *Ixodes dammini*. Emerg. Infect. Dis..

[8] Ebel GD (2010). Update on Powassan virus: Emergence of a North American tick-borne flavivirus. Annu. Rev. Entomol..

[9] Madison-Antenucci S, Kramer LD, Gebhardt LL, Kauffman E (2020). Emerging tickborne diseases. Clin. Microbiol Rev..

[10] Goddard, J., Varela-Stokes, A. Role of the lone star tick, *Amblyomma americanum* (L), in human and animal diseases. *Vet Parasitol*. 160(1-2):1–12 (2009). doi: 10.1016/j.vetpar.2008.10.089. 10.1016/j.vetpar.2008.10.089 19054615

[11] Abdelmaseih R, Ashraf B, Abdelmasih R, Dunn S, Nasser H (2021). Southern tick-associated rash illness: Florida’s Lyme disease variant. Cureus.

[12] Savage HM, Godsey MS, Lambert A, Panella NA, Burkhalter KL, Harmon JR, Lash RR, Aashley DC, Nicholson WL (2013). First detection of Heartland virus (Bunyaviridae: Phlebovirus) from field collected arthropods. Am. J. Trop. Med. Hyg..

[13] Savage HM, Burkhalter KL, Godsey MS, Panella NA, Ashley DC, Nicholson WL, Lambert AJ (2017). Bourbon virus in field-collected ticks, Missouri, USA. Emerg. Infect. Dis..

[14] Mitchell CL, Lin FC, Vaughn M, Apperson CS, Meshnick SR, Commins SP (2020). Association between lone star tick bites and increased alpha-gal sensitization: Evidence from a prospective cohort of outdoor workers. Parasit. Vectors..

[15] Whitten T (2019). Prevalence of *Francisella tularensis* in *Dermacentor variabilis* ticks, Minnesota, 2017. Vector-Borne Zoonotic Dis..

[16] Hecht JA, Allerdice MEJ, Dykstra EA, Mastel L, Eisen RJ, Johnson TL, Gaff HD, Varela-Stokes AS, Goddard J, Pagac BB, Paddock CD, Karpathy SE (2019). Multistate survey of American Dog Ticks (*Dermacentor variabilis*) for *Rickettsia* species. Vector Borne Zoonotic Dis..

[17] Lother SA, Haley L (2017). Tick paralysis. CMAJ.

[18] Sundstrom KD, Lineberry MW, Grant AN, Duncan KT, Ientile MM (2021). Little, SE Equine attachment site preferences and seasonality of common North American ticks: *Amblyomma americanum*, *Dermacentor albipictus*, and *Ixodes scapularis*. Parasit. Vectors.

[19] Saleh MN, Sundstrom KD, Duncan KT, Ientile MM, Jordy J, Ghosh P, Little SE (2019). Show us your ticks: A survey of ticks infesting dogs and cats across the USA. Parasit. Vectors..

[20] Tiffin HS, Skvarla MJ, Machtinger ET (2021). Ticks abundance and life-stage segregation on the American black bear (*Ursus americanus*). Int J. Parasitol. Parasites Wildl..

[21] Jang MS, Kim CM, Kim DM, Yoon NR, Han MA, Kim HK, Oh WS, Yoon HJ, Wie SH, Hur J (2016). Comparison of preferred bite sites between mites and ticks on humans in Korea. Am. J. Trop. Med. Hyg..

[22] Cull B, Pietzch ME, Gillingham EL, McGinley L, Medlock JM, Hansford KM (2020). Seasonality an anatomical location of human tick bites in the United Kingdom. Zoonoses Public Health.

[23] Okulova NM, Chunikhin SP, Vavilova VE, Maiorova AD (1989). The Location of the infecting tick bite and the severity of the course of tick-borne encephalitis. Med. Parazitol. (Mosk).

[24] Kiffner C, Lödige C, Alings M, Vor T, Rühe F (2011). Attachment site selection of ticks on roe deer, Capreolus capreolus. Exp. Appl. Acarol..

[25] Mysterud A, Hatlegjerde IL, Sørensen OJ (2014). Attachment site selection of life stages of *Ixodes ricinus* ticks on a main large host in Europe, the red deer (*Cervus elaphus*). Parasit. Vectors.

[26] Eisen L (2018). Pathogen transmission in relation to duration of attachment by Ixodes scapularis ticks. Ticks Tick Borne Dis..

[27] Piesman J, Mather TN, Sinski RJ, Spielman A (1987). Duration of tick attachment and Borrelia burgdorferi transmission. J. Clin. Microbiol..

[28] Hermance ME, Thangamani S (2018). Tick-virus-host interactions at the cutaneous interface: The nidus of flavivirus transmission. Viruses.

[29] Cook MJ (2014). Lyme Borreliosis: A review of data on transmission time after tick attachment. Int J Gen Med..

[30] Allan BF, Goessling LS, Storch GA, Thach RE (2010). Blood meal analysis to identify reservoir hosts for *Amblyomma americanum* ticks. Emerg. Infect. Dis..

[31] Mendell NL (2019). Detection of Rickettsiae, Borreliae, and Ehrlichiae in ticks collected from Walker County, Texas, 2017–2018. Insects.

[32] Williams SC (2021). Effective control of the motile states of Amblyomma americanum and reduced *Ehrlichia* spp. prevalence in adults via permethrin treatment of white-tailed deer in costal Connecticut, USA. Ticks Tick Borne Dis..

[33] Feder HM, Hoss DM, Zemel L, Telford SR, Dias F, Wormser GP (2011). Southern tick-associated rash illness (STARI) in the north: STARI following a tick bite in Long Island, New York. Clin Infect Dis..

[34] Ramirez-Garofalo JR, Curley SR, Field CE, Hart CE, Thangamani S (2022). Established populations of *Rickettsia parkeri-*infected *Amblyomma maculatum* ticks in New York City, New York, USA. Vector Borne Zoonotic Dis..

[35] Durden LA, Luckhart S, Mullen GR, Smith S (1991). Tick infestations of white-tailed deer in Alabama. J. Wildl. Dis..

[36] Fryxell RTT, Steelman CD, Szalanski AL, Kvamme KL, Billingsley PM, Williamson PC (2012). Survey of Borreliae in ticks, canines, and white-tailed deer from Arkansas, USA. Parasit. Vectors.

[37] Harris PA, Taylor R, Thielke R, Payne J, Gonzalez N, Conde JG (2009). Research electronic data capture (REDCap). A metadata-driven methodology and workflow process for providing translational research informatics support. J Biomed Inform..

[38] Harris PA, Taylor R, Minor BL, Elliott V, Fernandez M, O’Neal L, McLeold L, Delacqua G, Delacqua F, Kirby J, Duda SN (2019). The REDCap consortium: The REDCap consortium: Building an international community of software partners. J. Biomed. Inform..

