# Supplementary material for: Human attachment site preferences of ticks parasitizing in New York
Source: Sci Rep. 2022 Dec 3;12:20897. doi: 10.1038/s41598-022-25486-7 (PMC9719493; doi:10.1038/s41598-022-25486-7)
Supplement: Supplementary file 1 — Supplementary Information. [file 41598_2022_25486_MOESM1_ESM.docx]

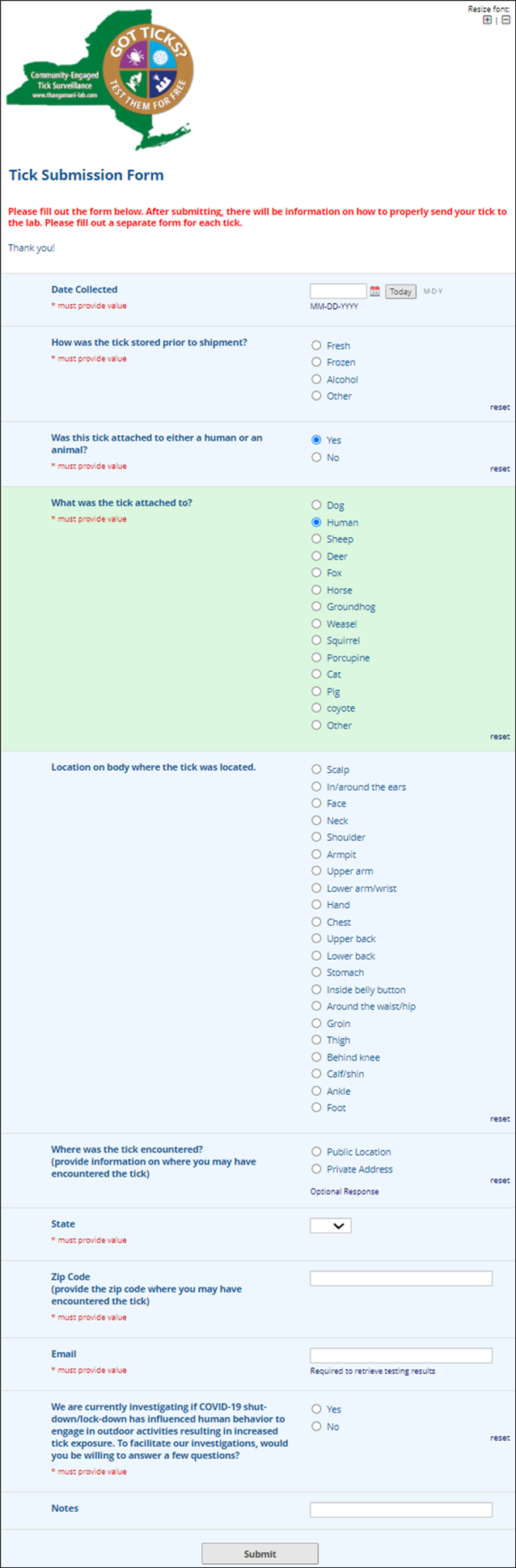
**Supplementary Figure 1:** An image of the online questionnaire utilized for the Community engaged passive surveillance program. Using this survey, tick submitters are able to provide additional information about where the tick was found in terms of location, host, and the date it was collected. This information was later filtered to select only ticks found biting humans from April-December 2020.
